# Toxoplasma Lymphadenopathy: A Comparative Diagnostic Assessment of Clinical, Serological and Histopathological Findings

**DOI:** 10.22038/IJORL.2023.64479.3205

**Published:** 2023-05

**Authors:** Samah Abbas Hammadi, Abbas Jaafar Khaleel Al-Anbari, Bassam Maddah Al-Alosi

**Affiliations:** 1 * Otolaryngologist, College of Medicine, Al-Nahrain University, Iraq.*; 2 * Consultant Cardiovascular and Thoracic Surgery, College of Medicine, Al-Nahrain University, Baghdad, Iraq.*; 3 *College of Medicine, University of Anbar, Iraq.*

**Keywords:** Biopsy, ELISA, Toxoplasma Gondii, Lymphadenopathy, Real-time PCR, Serology, Lymph node

## Abstract

**Introduction:**

Toxoplasma Gondii (TG) is a zoonotic protozoan with extensive symptomatology. Toxoplasmic lymphadenopathy is considered an affirmative sign and is proved by a biopsy of the enlarged nodule. This study was conducted to compare the clinical, serological, and histopathological findings for the diagnosis of toxoplasmic lymphadenopathy.

**Materials and Methods:**

This study involved biopsy examinations from twelve cases with TG lymphadenopathy. ELISA serological tests were performed for TG specific IgM and IgG immunoglobulins. PCR was done to ratify the results obtained by ELISA.

**Results:**

The ages of the patients ranged from 15 to 48 years (mean=27.8). Most of the cases are male n=8(66.7%), while female n=4(33.3%). The asthenia was not only the most frequent clinical presentation (83.3%), but it also last longer. All cases had a positive biopsy. Eight (67.7%) cases revealed seropositivity. Two of them had positive PCR in those who were positive IgM, suggesting that the infection was acute. Six (50%) cases revealed positive IgG tests, while those with negative serology were 4(33.3%). The site of lymph nodes involvement had been assessed and mostly cervical (91.6%).

**Conclusion:**

The histopathological results yielded 100% positive findings, thus biopsy was very important in the diagnosis and differential diagnosis of lymph nodes enlargement. The chronic phase of toxoplasmosis does not show the protozoa in the blood causing an absent DNA band for amplification of the PCR cycles, which could explain the lack of bands particular for TG. A negative serological test does not exclude toxoplasmic lymphadenitis, especially in immune-compromised patients.

## Introduction

A wide range of living animals can be infected by Toxoplasma Gondii (TG), which is a widely prevalent intracellular protozoan. It can virtually infect all blooded warm animals. The Felidae family are the final host that excretes oocysts. An infected cat excretes 300,000 to approximately 100 million oocysts in up to 20 days without disease in it. Human being acquires infection by the ingested oocyst-contaminated nutrients or water, in the course of organ transplantation, laboratory accidents, blood components transfusion, and congenitally (1). The TG parasite can remain dormant inside tissues all over the host. *TG *belongs to the phylum Apicomplexa, the family *Sarcocystidea*. It was first observed in rodents in 1908. There are three biological forms of TG, which are oocysts, bradyzoites (or tissue cysts), and tachyzoites. The oocysts comprise sporozoites, which represent the final products of the sexual cycles in the intestinal epithelial cells of their final host (cats) that form oocysts being shed in the fecal materials. The asexual invasive TG form (i.e. tachyzoite), is reproduced principally inside any nucleated cells (2). The bradyzoite or tissue form can persist dormant in the tissues for years. The bradyzoites can arise whenever the host immune mechanisms deteriorate and present clinically in form of acute toxoplasmosis (3).

Toxoplasmosis is derived from the Greek word ^"^ Toxon^"^ which means bow as a unique crescent-shaped trophozoite, half the size of RBC. Approximately 25-30% of the world’s human population is infected but this varies greatly between and within countries (3). The human infection by TG is found universally, in a diversity of environments or socioeconomic conditions. Infection is high in hot, humid conditions and low altitudes. Cysts are highly resistant, survive even up to 18 months. High seroprevalence in countries where undercooked meat is eaten like France and Central America (4). The clinical manifestation of TG infection is influenced by the age and immunity of the patients (3). It can produce severe fetal neuronal or ocular diseases during intrauterine life. Ocular pathologies consist of chorioretinitis, anterior uveitis, and other presentations. In most immunocompetent patients, the primary infection is generally asymptomatic (3). Nevertheless, patients may have pyrexia, malaise, chills, lymph node enlargement, sweating, headaches, or muscle ache. Since less than 10% of infected patients exhibit symptoms, lymphadenopathy arises in 10-20% of acute infections and might be associated with constitutional manifestations (5, 6). While those who are immunocompromised, a multisystemic form can develop, with multiorgans involvement, like the cardiac, CNS, lungs, and ocular signs (7).

The current study was conducted to compare clinical presentation, serological test, and histopathological examination in the conformation of the diagnosis of toxoplasmic lymphadenitis.

## Materials and Methods

*Subjects*

Twelve cases suspected to have toxoplasmic lymphadenitis were evaluated clinically, serology and histopathology during 8 years’ period, from January 2012 to January 2020. The mean ages of the TG lymphadenopathy patients were 27.8±3.8 year, and the males represented 66.7% (n=8).

Medical past histories of all 12 cases, including age, sex, clinical presentation, location of lymphadenopathy, state of immunity of patients, and ultrasound findings were obtained. 

*Biopsy, serology, and PCR investigations*

All the 12 cases underwent lymph node biopsy along with blood sampling of (2ml) were drained into separate blood test tubes and the sera were centrifuged and separated. The serological tests were performed for TG (IgM and IgG) by a specific "Human TG ELISA Kit (Biocompare®, MyBioSource-USA)". PCR was tested on only eight blood samples that revealed seropositive outcomes.

*Ethical Issues*

An informed consent was obtained from each subject or subject’s guardian, before being involved in this work. The whole study was approved by the authorities of local health institution of Baghdad health directorate.

*Molecular investigations*

Whole blood samples were used for PCR tests accomplishment, which was achieved according to the company instructions using PCR kit, "Sacace^®^ Biotechnologies, Italy". The PCR tests used the real-time PCR instrument from "Rotor-Gene Q - QIAGEN^®^, England". 

## Results

**Fig 1:**
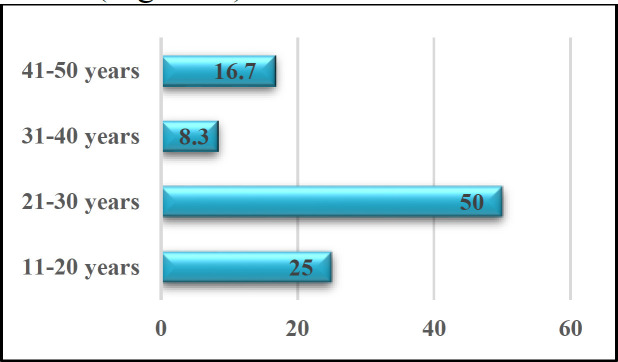


***The ages of the patients range from 15 years to 48 years, with a mean age of 27.8±3.8) years. Half of the patients were within their 2*** ^nd^ ***-3*** ^rd^ ***decades of life, 1/4*** ^th^ ***were within their 1*** ^st^ ***- 2*** ^nd^ ***decade of life. 16.7% of the patients were in their 5*** ^th^ ***decade, and the rest were in the fourth decade (*** Figure-1 ***).***

**Fig 2:**
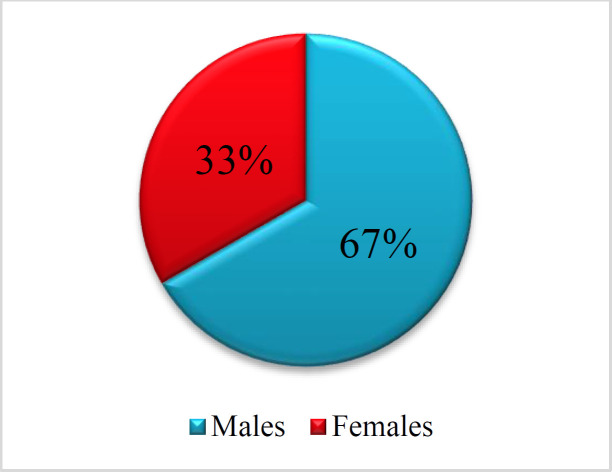
Sex distribution of the studied patients

The sex distribution of the patients was shown in the figure-2. Most of the cases were the males, n=8 (66.7%), while the females' number was 4 (33.3%).

**Fig 3:**
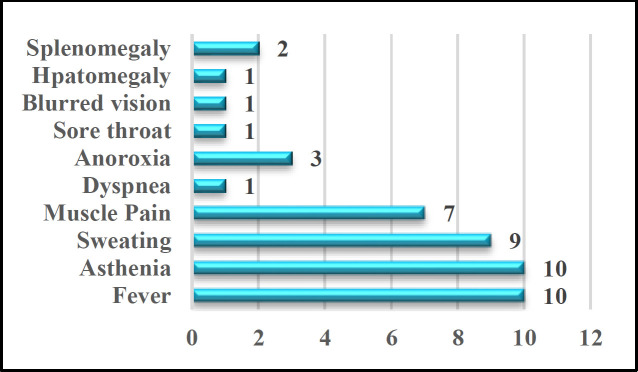
Clinical manifestations of toxoplasmic lymphadenitis among the included cases (n-12)

The most recorded clinical signs and symptoms of toxoplasmic lymphadenitis among the studied patients were exhibited in figure-3. The commonest manifestations were asthenia and fever which were observed in 10 (83.3%) of them. The next most common signs were sweating and myalgia [9 (75%) and 7 (58.3%)], respectively. The remaining uncommon signs included anorexia, hepatomegaly, splenomegaly, sore throat, blurred vision, and shortness of breath.

**Fig4:**
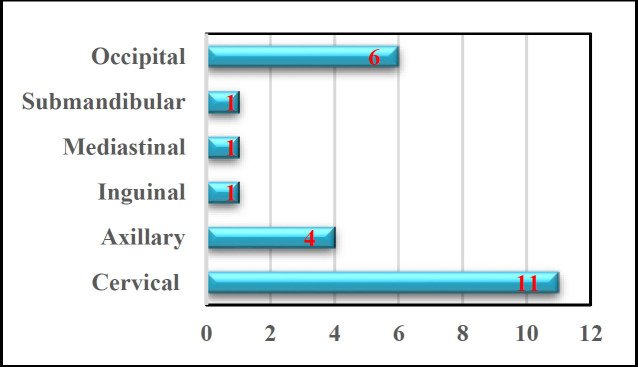


The site of lymph nodes involvement had been assessed and mostly cervical with 91.6%. The other groups that were involved had been shown in the figure-4.

Four patients who revealed negative serological tests had a previous history of immune suppression; three of them were on chemotherapy for treatment of malignancy and the fourth patient was accidentally discovered to be infected with the human immunodeficiency virus (Table-1).

**Table 1:** The distribution of the patients’ serology according to their history

**Past medical history**	**Serology tests**	**Number**	**Percent**
No previous illness	Positive	8	66.7
Immune compromised patients			
History of chemotherapy	Negative	3	22.2
Human immunodeficiency virus	Negative	1	11.1

The histopathological examination had been done from the enlarged lymph nodes and the results with 100% conformation of diagnosis of toxoplasmic lymphadenitis. Serological tests had been done regarding positive IgG, and IgM antibodies with n=6 (50%), n=2 (16.7) respectively and PCR was positive to those IgM positive, while those with negative serology were n=4 (33,3%) as shown in (table-2).

**Table 2:** Distribution of the studied patients according to their positive results including, histopathology, serology (IgG and IgM), and PCR finding

	**Number of positive results**	**%**
Histopathology	12	100
IgG	6	50
IgM	2	16.7
PCR	2	16.7

## Discussion

The presentations of toxoplasmosis have two clinical forms: either congenital or acquired (8, 9). The main characteristics of the congenital TG infection are chorioretinitis, hydrocephaly, convulsive fits, and cerebral calcifications of neonatal babies. The manifestations of congenital TG may be observed at birth or will be seen later in life (10,11).

The acquired TG form can be further divided into lymphadenopathic (the commonest form) and the disseminated type (12, 13). Acquired TG infections can be presented at any age interval, but the peak prevalence is in the 2^nd^ and 3^rd^ decades (4). McCabe et al. found in their study mean age was 26 years (12). In the current study, the ages of the patients range from 15-48 years of age with a mean age reaching 27.8years. In his study, Beverley displayed that around 30% of TG lymphadenopathy subjects were either children or adolescents (14). The current study shows that 3 patients, from the total 12 cases that were studied, are young adolescents with 25 %. 

The head and neck lymphadenopathy of TG infection is a common sign and may be associated with different pathological etiologies. Localized and/or generalized lymphadenopathy may be a presenting sign of several diseases such as lymphoma (15, 16), localized infections (17, 18), infectious mononucleosis, cytomegalovirus, cat-scratch disease, sarcoidosis, toxoplasmosis, non-Hodgkin's, and Hodgkin's metastatic malignant disease (15, 16), tuberculosis (17, 18), tularemia, brucellosis, dermatopathic adenitis, rubella and others (6). It has been expected that up to 15% of unexplainable lymphadenopathies have resulted from toxoplasmosis, commonly affecting the lymph nodes of the cervical areas (19, 20). TG infection should be regarded in the differential diagnosis of any lymph enlargement in the head and neck. Because of that histopathological examination should be considered to exclude malignancy and establish the diagnosis. Lymphadenopathy is a classic and infrequently a single presenting sign of the acquired TG infection; usually soft, movable, and occasionally painful (20). Sumi Y. found in his study that cervical adenopathy was reported in the dorsal cervical area (82%), then axillary (35%), inguinal (19%), and anterior thoracic wall (8%). While RA. Durlach et al., at Buenos Aires, Argentina, found that the cervical lymph node involvement was (92.5%), the occipital was (73.3%), the axillary was (37.5%), and the inguinal lymph nodes involvement was (11.6%) (21). The current study shows that the posterior cervical group of lymph nodes is mostly involved with (91.6%), the axillary group of lymph nodes involvements is (33.3%), and the inguinal lymph nodes involvements are (8.3%). 

The clinical manifestations associated with toxoplasmosis infections are nonspecific and frequently asymptomatic; they may include asthenia, low-grade fever, fatigue, and lymphadenopathy. 

The current study shows that the asthenia was not only the most frequent clinical presentation (83.3%), but it also last longer, with a duration reached 10 months in one of the patients. However, Jones et al. described an incidence rate of 40% (22). While Durlach RA. et al. reported a rate of 69% and found that asthenia might continue for months peaked up to one year (21).

Fever and sweating, which are other symptoms to be expected in infectious diseases, were observed in the current study (83.3% and 75%), respectively, and the fever was mostly low grade and lasts from 3-7 days. However, Jones et al. in his study reported the fever in 37% of the TG infection (22) and Durlach RA. et al. reported a rate of 45% among toxoplasmosis patients (21). In another study that included TG infected subjects, the incidence of high temperature was 74% (12). 

Other manifestations that have been found in the current study include myalgia (58.3%), anorexia (25%), sore throat (8.3%), dyspnea (8.3%), hepatomegaly (8.3%), splenomegaly (16.6%), and blurred vision (8.3%). The patient who presented with blurred vision had been diagnosed by an ophthalmologist with chorioretinitis.

During TG infection, the blood investigations might demonstrate a trivial monocytic leukocytosis and lymphocytosis. The precise identification of TG lymphadenitis can be proven by histopathological findings and further confirm by specific serology studies. The serologic investigations of TG can include immunoglobulins assay of IgE, IgA, IgG, and IgM (23).

The foremost serological tests for the diagnosis of a suspected case of TG infections are IgG and IgM. IgM immunoglobulins can be measured at the end of the second week after TG infection, reach its topmost in one month, and fall thereafter, to become untraceable during the next 6-9 months. A single plasma titer of 1:80 or greater IgM is diagnostic; nevertheless, a negative titer does not exclude TG infection (5). 

Generally, IgG immunoglobulins become positive 2-3 weeks after TG infection, attain the highest level at 1-2 months later, and continue positively for life (24). Iddawela W. et al. found in their study of 17 patients diagnosed clinically and histopathologically as toxoplasmic lymphadenopathy that only 35.3% had positive antibodies (25). While Durlach RA. et al. In a study of 120 cases of TG lymphadenopathy reported that IgM immunosorbent agglutination assay was positive in 98%, which is highly indicative of recent infection (21). The current study shows that IgG antibodies are positive in 50%, which indicates chronic infection, IgM antibodies are positive in 16.7%, which indicates recent infection. The current study shows that those patients with negative serological tests are already with a history of immune suppression consistent with a case report published recently (13). In later decades, the polymerase chain reaction (PCR) technique is being described progressively as a potent diagnostic tool. A preceding study revealed that PCR of peripheral blood samples was beneficial in the diagnosis of cerebral TG infection among AIDS patients (26). A swell, the PCR was effectively practiced on amniotic fluid samples for antenatal diagnosis of the congenital form of toxoplasmosis (27). Despite that, the prolonged TG infection does not reveal the organism in the blood resulting in poor DNA amplification for PCR test, which in turn clarifies the lack of DNA bands particular for TG in this study, i.e. 6-negative PCR results (25). The current study finds that patients with IgM positive test get positive PCR test in 1.7%, which indicates recent infection. Consequently, it is suggested that the ELISA serology tests be utilized to diagnose TG infection besides the verification of lymph node biopsy (25).

Samples of lymph node tissue are frequently done to discriminate cancer from TG infection in the case of lymphadenopathy. The histologic variations in TG lymphadenitis are unique and often characteristic (28, 29). A distinctive histopathological form of TG lymphadenitis showed numerous subcapsular microgranulomas (30). Further, histopathologic triad present in TG lymph node infection are as follows; 1) follicular hyperplasia, 2) masses of proliferative epithelioid histiocytes besides a mixed lymphocytic and immunoblastic cell cluster 3) cortical and marginal sinuses filled with epithelioid cells. The diagnosis of TG lymphadenitis is proven by histopathological studies and confirmed by serologic studies (31). There is a prominent reactive follicular hyperplasia along with abundant mitoses. As well, the germinal centers exhibit karyorrhectic debris. There are groups of histiocytes in the paracortical interfollicular and cortical areas, and the histiocytes typically impinged on and blur the borders of the reactive follicles (28, 29). The microscopic appearance is very significant in distinguishing this lesion from riskier malignant lymphomas, like "Hodgkin's lymphoma". Initially, in toxoplasmosis, the architecture of the lymph nodes is preserved. Next, the proliferating cells display a normal mitotic process, and merely among them does any substantial necrosis seen. Moreover, the giant cells of Hodgkin's disease are not observed. Lastly, the infiltrating cells, characteristic for inflammation, in TG is also involve the capsule and spread out of the node (32). When the history of the patients was re-evaluated, the current study finds that all cases have a common history of eating the undercooked liver of sheep and drinking municipal water supply. As compared to Choi WY et al. finding in his report of 5 out of 11 Korean militaries who consumed the uncooked domestic pig livers, presented with generalized lymph enlargement (33). As well, the metropolitan water supply was the cause of a previous outbreak of TG infection in Victoria, Canada (34).

## Conclusion

The study revealed that histopathological findings produced 100% (12/12) positive results, thus biopsy was very important in the conformation of the diagnosis and exclude other causes of lymph nodes enlargement. Chronic infection does not have the organism in the bloodstream resulting in the absence of DNA for PCR amplification which in turn explains the absence of bands specific for T. Gondii. A negative serological test does not exclude toxoplasmic lymphadenitis, especially in immune-compromised patients. Because toxoplasmic lymphadenitis is found worldwide, surgeons and physicians have to keep it in mind in any patient with lymphadenopathy.
